# Safety of Oral Amphetamine Administered during Positron Emission Tomography Scans in Medically Screened Humans

**DOI:** 10.1371/journal.pone.0140647

**Published:** 2015-12-14

**Authors:** Lora D. Weidner, Antonio Paris, W. Gordon Frankle, Rajesh Narendran

**Affiliations:** 1 Department of Radiology, University of Pittsburgh, Pittsburgh, Pennsylvania, United States of America; 2 Department of Psychiatry, University of Pittsburgh, Pittsburgh, Pennsylvania, United States of America; Centre for Addiction and Mental Health, CANADA

## Abstract

Changes in endogenous dopamine levels can be detected in humans using positron emission tomography scans by measuring the amount by which a specific D_2/3_ radioligand is displaced. In some cases, a challenge drug such as amphetamine is introduced to increase the amount of dopamine released into the synaptic cleft. Although intravenous amphetamine is often utilized, oral amphetamine has been shown to be just as effective in increasing endogenous dopamine levels. Based on our own use of oral amphetamine as a challenge drug, we have retroactively reviewed our study charts to determine the cardiovascular safety of 0.5 mg kg^-1^ oral d-amphetamine. Of 172 amphetamine administrations in 144 individuals, only 2.8% of subjects experienced any transient adverse effects. In addition, we found no clinically relevant differences in increases of vital signs between healthy controls and patients. We therefore reaffirm the safety of 0.5 mg kg^-1^ oral amphetamine in subjects previously screened for cardiovascular risk factors.

## Introduction

Over the past two decades, positron emission tomography (PET) and single photon emission tomography imaging techniques have been used to measure changes in endogenous dopamine levels in humans. Disturbances in dopamine transmission are implicated in a multitude of neuropsychiatric disorders including drug addiction, schizophrenia, and attention deficit hyperactivity disorder. These fluctuations in extracellular concentrations can be indirectly determined with PET imaging *in vivo* by measuring the binding potential of a radiolabeled D_2/3_ receptor antagonist before and after a drug challenge [1]. In these specific cases, a drug challenge is defined as a drug that is known to increase the amount of dopamine released into the synaptic cleft, such as cocaine, amphetamine, or methylphenidate. This allows researchers to not only measure differences in baseline dopamine release, but to also determine the extent to which the increase of dopamine after a drug challenge changes between patient groups.

Although a potent central nervous system stimulant, the use of amphetamine to increase extracellular dopamine for PET imaging has been well validated, with acute administrations in humans considered relatively safe. When comparing oral versus intravenous routes of administration however, intravenous amphetamine can lead to rapid changes in cardiovascular vital signs while the inherently slower absorption of oral amphetamine decreases the risk of inducing unwanted side effects [2–5]. The same applies to oral versus intravenous methylphenidate, in which the former results in decreased behavioral effects [6]. The administration of oral amphetamine has been found to be just as effective in modulating dopamine activity when compared to intravenous amphetamine [2, 5]. Nonetheless, it remains important to reaffirm the safety of oral amphetamine administrations in humans to ensure there are as few cardiovascular risks to subjects as possible.

Herein the authors retroactively examine the peripheral effects of oral amphetamine across 172 acute administrations performed by the Psychiatric Molecular Imaging Program, University of Pittsburgh in order to confirm the safety of this challenge drug in humans for future PET imaging studies. To this end, subject charts were reviewed for measures such as blood pressure (BP) and heart rate (HR) to determine the extent to which amphetamine administration affects cardiovascular systems.

## Methods

The studies included in this analysis were approved by the Institutional Review Board (IRB) of the University of Pittsburgh Medical Center, and were conducted in accordance with the Belmont Report. For each study, a signed informed consent was obtained from each subject. A total of 172 subject charts were reviewed for cardiovascular vital sign measurements, which included BP and HR. Focus was placed on three time-points to make analysis simpler: baseline (taken prior to amphetamine administration), peak (the highest recording taken after amphetamine administration), and discharge (final set of vital signs taken that day). Inclusion criteria required subjects to have had at least one oral d-amphetamine administration (0.5 mg kg^-1^). If a subject received more than one amphetamine administration, all administrations were counted as separate events.

The following methods pertain to the studies include in the analysis. A physical screening was conducted to eliminate subjects with a weight greater than 113 kg, a baseline BP above 140/90, and/or those who had an abnormal EKG. On the day of the PET, vital signs were monitored continuously from baseline up to at least 250 minutes post amphetamine administration. Oral d-amphetamine (Dexedrine) was dispensed to the subject in 2.5 mg pills to equal 0.5 mg kg^-1^ of total bodyweight under medical supervision, and the subjects remained under medical supervision for the remainder of the study. Amphetamine administration occurred 3 hr prior to the post-amphetamine PET scan. Discharge required subject’s BP to have returned to less than 20 mm Hg above their baseline reading for systolic (SBP) and diastolic BP (DBP) or less than 150/90 (whichever reading is lower), HR to within 20 beats per min (bpm) of baseline, and have a normal EKG. Additionally, there could be no remaining subjective effects or continuing adverse events (as defined by the FDA 21CFR312.32) related to the amphetamine.

The studies incorporated in this analysis include administrations in healthy control participants (n = 106), as well as patients with attention deficit hyperactivity disorder (n = 6), alcoholism (n = 24), cocaine addiction (n = 6), eating disorders (n = 7), marijuana addiction (n = 10), and schizophrenia (n = 13). Statistical significance was evaluated by a repeated measures ANOVA for all data except for mean arterial pressure for which a one-way ANOVA was used. Both calculations were followed by the Bonferronni post *t*-test (α = 0.05) and were performed using SPSS (Armonk, New York).

## Results

Charts were reviewed for 172 administrations of amphetamine in 144 individuals (90 females; ages 18–55). From this, four subjects (2.8%) were given lorazepam (n = 3, 1–5 mg) or labetalol (n = 1, 200 mg) for an increased HR, in which all incidents resolved without sequelae. The highest vital signs observed was a BP of 199/117 in one subject and a HR of 150 bpm in another. No other amphetamine related adverse events occurred. Of the 144 subjects, several subjects received more than one amphetamine administration due to the study protocol in which they were enrolled. The vital signs from these scans were originally included as a between-subjects factor but they showed no significant difference (SBP: *P* = 0.577, DBP: *P* = 0.432, HR: *P* = 0.072) and had no major impact on the results. Therefore we chose to report all 172 administrations as their own unique instance.

### Systolic Blood Pressure

**Fig 1:**
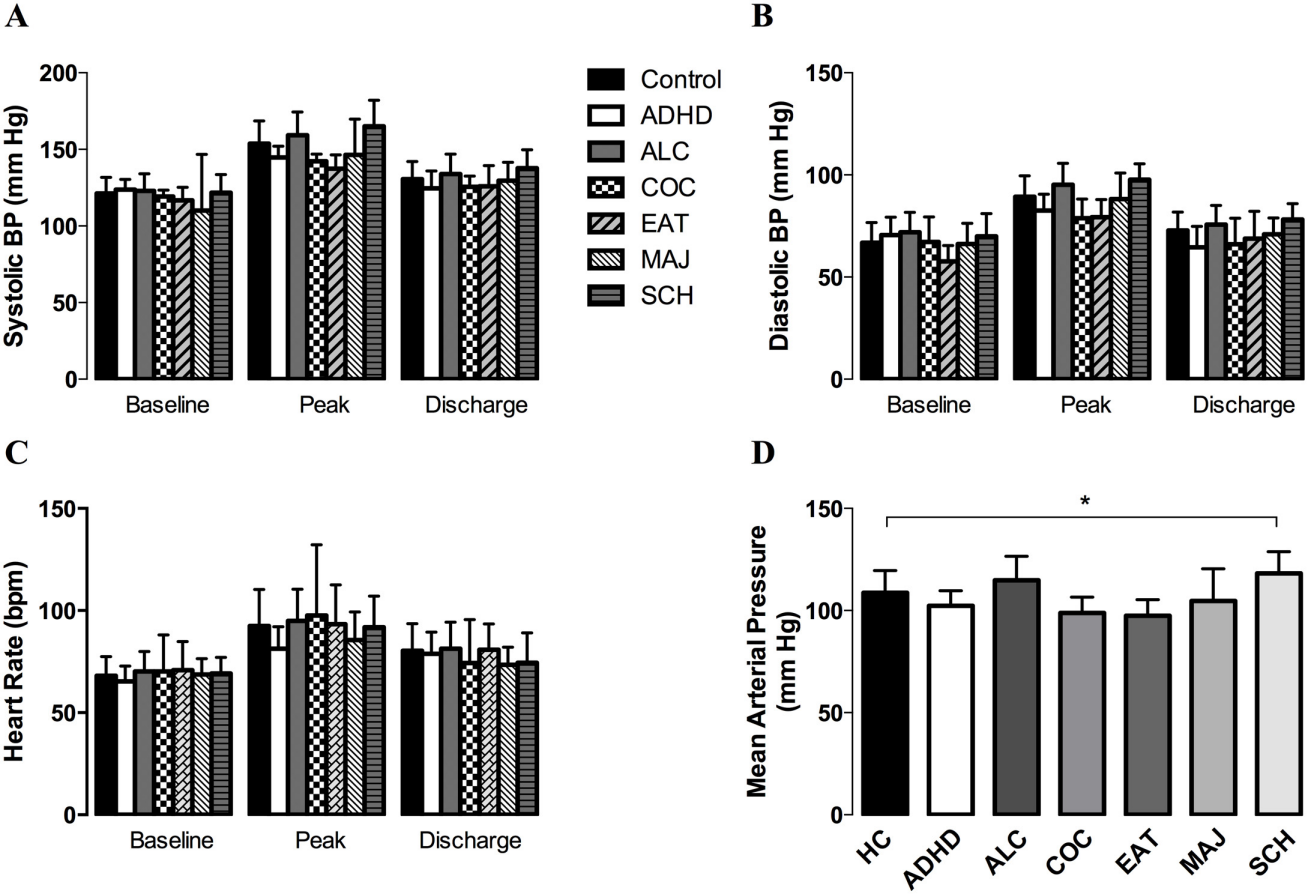
Vital signs on day of PET scan. (A) Systolic blood pressure, (B) diastolic blood pressure, (C) and heart rate at baseline, peak, and discharge time points for all subjects. (D) Mean arterial pressure during the amphetamine administration for all subjects. Abbreviations: ADHD, attention deficit hyperactivity disorder; ALC, alcoholism; COC, cocaine addiction; EAT, eating disorders; MAJ, marijuana addiction; SCH, schizophrenia; bpm, beats per minute. *, *P* < 0.05 by a one-way ANOVA followed by the Bonferronni post *t*-test (α = 0.05).

A repeated measures ANOVA for baseline, peak, and discharge readings revealed a significant effect of amphetamine, *F*(2,170) = 396.8, *P* < 0.0001. Compared to baseline, the average peak recording across all subjects increased by 32.7 ± 1.18 mm Hg (*P* < 0.0001). Discharge was 10.2 ± 1.01 mm Hg higher than baseline for all subjects (*P* < 0.0001). The average peak increase did not differ significantly between healthy controls (32.4 ± 1.36 mm Hg, Table 1) and patients (33.4 ± 2.17 mm Hg) when all patient groups were analyzed together. A comparison of each patient subtype to the healthy control group revealed no significant differences (Fig 1).

**Table 1:** Oral versus Intravenous Amphetamine in Healthy Subjects.

	Oral Amphetamine (n = 106)		Intravenous Amphetamine (n = 24)*	
Vital Sign	Mean	SD	Mean	SD
**Systolic blood pressure**				
Mean baseline (mm Hg)	121.3	10.4	119.5	12.2
Average peak (mm Hg)	153.7	14.9	180.8	18.3
Average time of peak (min)	103.2	54.2	7.4	7.4
**Diastolic blood pressure**				
Mean baseline (mm Hg)	66.8	10.0	71.3	9.4
Average peak (mm Hg)	89.4	10.2	102.5	9.7
Average time of peak (min)	98.8	59.9	7.9	11.2
**Heart rate**				
Mean baseline (mm Hg)	68.1	9.4	64.1	8.7
Average peak (mm Hg)	92.4	17.9	81.4	14.6
Average time of peak (min)	122.8	97.5	18.9	19.0

* Data from Martinez et al., 2007

### Diastolic Blood Pressure

A repeated measures ANOVA for baseline, peak, and discharge readings revealed a significant effect of amphetamine, *F*(2,170) = 372.9, *P* < 0.0001. Compared to baseline, the average peak recording across all subjects increased by 22.2 ± 0.85 mm Hg (*P* < 0.0001). Discharge was 5.23 ± 0.83 mm Hg higher than baseline for all subjects (*P* < 0.0001). The average peak increase did not differ significantly between healthy controls (22.4 + 1.11 mm Hg, Table 1) and patients (21.7 + 1.31 mm Hg) when all patient groups were analyzed together. A comparison of each patient subtype to the healthy control group revealed no significant differences (Fig 1.)

### Heart Rate

A repeated measures ANOVA for baseline, peak, and discharge readings revealed a significant effect of amphetamine, *F*(2,170) = 206.8, *P* < 0.0001. Compared to baseline, the average peak recording across all subjects increased by 23.6 ± 1.16 bpm (*P* < 0.0001). Discharge was 10.8 ± 0.96 bpm higher than baseline for all subjects (*P* < 0.0001). The average peak increase did not differ significantly between healthy controls (24.4 ± 1.57 bpm, Table 1) and patients (22.4 ± 1.69 bpm) when all patient groups were analyzed together. A comparison of each patient subtype to the healthy control group revealed no significant differences (Fig 1).

### Mean Arterial Pressure

Mean arterial pressure was calculated using the peak post-amphetamine BP for each subject. A one-way ANOVA revealed the patients with schizophrenia to have a significantly higher mean arterial pressure (118.2 ± 10.6 mm Hg) when compared to healthy controls (108.7 ± 10.9 mm Hg, *P* < 0.05, Fig 1). No other statistical significance was found for the remaining patient sub-groups.

**Fig 2:**
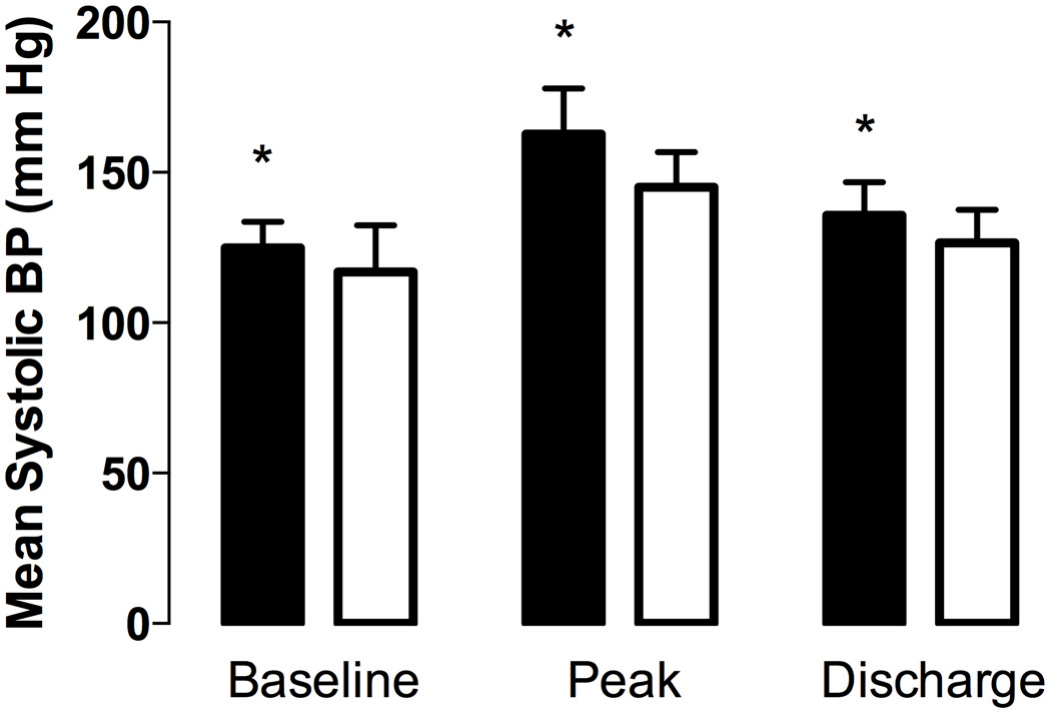
Gender differences in systolic blood pressure. Systolic blood pressure readings from males (black bars) and females (white bars) across three separate measurements. The analysis includes both healthy controls and patients. *, *P* < 0.0001 by a repeated measures ANOVA followed by the Bonferronni post *t*-test (α = 0.05).

A *post hoc* comparison of gender revealed males to have a statistically higher SBP than females at baseline (males = 125.0 ± 8.6 mm Hg, females = 116.9 ± 15.5 mm Hg, P < 0.0001, Fig 2), peak (males = 162.7 ± 15.2 mm Hg, females = 145.1 ± 11.7 mm Hg, P < 0.0001, Fig 2), and discharge (males = 135.74 ± 11.0, females = 126.6 ± 11.0, P < 0.0001, Fig 2) measurements. In addition, males had a higher DBP than females at baseline (males = 69.8 ± 9.5 mm Hg, females = 65.4 ± 10.4 mm Hg, *P* < 0.01, Fig 2) and peak (males = 93.7 ± 11.5 mm Hg, females = 86.1 ± 8.9 mm Hg, *P* < 00001, Fig 2) measurements. There were no differences in HR between genders at any time point.

## Discussion

Our results confirm that administration of oral amphetamine (0.5 mg kg^-1^) during a PET imaging study is safe in persons who have been previously screened for cardiovascular risk factors. Other than a small percentage of subjects who experienced transient adverse events, the majority of subjects displayed a typical peripheral response to the stimulant. Nonetheless, it is imperative that future subjects be monitored closely for changes in cardiovascular vital signs when exposed to any amphetamine dose.

Many groups have utilized either oral methylphenidate or amphetamine for the purpose of measuring changes in dopamine release, however only a few groups have included changes in vital signs in their results. Nonetheless, the extant literature reveals similar increases in peripheral vital signs in healthy control subjects at comparable time points to our studies. Administration of 0.5 mg kg^-1^ oral amphetamine to subjects (n = 8) resulted in a peak SBP of approximately 150 mm Hg 2 hr post dosing [7]. In a similar fashion, 30 mg oral amphetamine (n = 12) led to an average increase in SBP from baseline of 20 ± 2.1 mm Hg 2 hr post administration [2], while intake of either 25, 30, or 35 mg (0.38–0.45 mg kg^-1^) oral amphetamine (n = 9) increased SBP by 17 ± 2.5 mm Hg when measured 90 min post administration [8]. These results are in agreement with our observed changes in SBP, which suggests good reproducibility in the vital signs of healthy controls in response to oral amphetamine.

Comparatively, intravenous injections of amphetamine have resulted in higher increases in BP in a shorter amount of time. In healthy controls, administration of 0.3 mg kg^-1^ amphetamine raised mean SBP by 42 ± 1.6 mm Hg (n = 8) [9], 43 ± 13 mm Hg (n = 7) [10], 61.3 ± 2.2 mm Hg (n = 24) [11], and 41 ± 14 mm Hg (n = 15) [12]. Similar increases were also observed in cocaine patients (59.7 ± 2.1 mm Hg, n = 24) [11] and schizophrenics (36 ±12 mm Hg; n = 15) [12]. Where reported, peak increases in SBP occurred within 25 min after injection compared to 102.2 ± 53.3 min after oral administration in our studies. Of these studies, Martinez and colleagues provided the most comprehensive data on the effects of intravenous amphetamine on vital signs, to which we have compared our healthy control data (Table 1) [11]. From this it is clear that oral amphetamine results in a lower peak in systolic and DBP across a longer period of time compared to intravenous amphetamine.

An earlier study conducted by our group found that 24 administrations of 0.5 mg kg^-1^ oral amphetamine in 12 healthy controls resulted in a mean peak SBP of 160 ± 13 mm Hg [5]. In the recently expanded cohort of n = 144 administrations, we observed the mean peak SBP to be slightly lower (153.5 ± 16.1 mm Hg). This further highlights the safety of oral amphetamine given that we possess data from a large cohort, which includes multiple patient subtypes. However the administration of amphetamine to individuals that have been screened for cardiovascular health is not without risk. Four subjects did receive medical intervention for a prolonged increased HR (HR did not return to less than 20 bpm from baseline at time of discharge). These subjects were given either labetelol or lorazepam, and remained under medical supervision until their HR returned to baseline. Subsequently they returned for a follow-up in which their vital signs and EKG were found to be normal. Although four subjects is a small percentage of the total number of participants (144), it still emphasizes the need for medical supervision when performing amphetamine challenge studies.

One limitation of this study was the number of variables used to determine cardiovascular safety after amphetamine administration. This limits our definition of “cardiovascular safety” to refer only to HR, BP, and cardiac rhythm documented with an EKG (performed at a single time point prior to discharge). For example, we cannot rule out the fact that abnormal cardiac rhythm may have been documented more often if we had performed continuous Holter Monitoring in subjects following amphetamine administration. In addition, we did not include data on subject’s subjective response after amphetamine. This data set is comprised of vital sign recordings from several different protocols, not all of which included surveys or questionnaires to capture the psychological (subjective) experiences of amphetamine in individuals. Nevertheless, none of the subjects reported any subjective adverse events (neurological or cardiac) that required medical intervention. We were also unable to detect any differences in peripheral response to amphetamine between patient sub-groups. Although this is not all that surprising, especially given a relatively low “n” for some groups, a recent study reported abnormal cardiovascular responses to oral amphetamine (0.4 mg/kg) in pathological gamblers (n = 12) as compared to healthy controls [13].

Based on the multitude of amphetamine administrations performed in our lab and others, we conclude 0.5 mg kg^-1^ oral amphetamine to be safe for the purpose of conducting PET imaging studies in healthy controls and patients who have been carefully screened to eliminate physiologic risk factors. Further research utilizing oral amphetamine (or methylphenidate) during PET imaging scans are crucial for understanding differences in dopamine transmission between patients and healthy controls, and the hope is that future groups will implement this paradigm with the knowledge that 0.5 mg kg^-1^ amphetamine can be administered safely under medical supervision.

## Supporting Information

S1 Table Raw data of vital signs. Systolic blood pressure, diastolic blood pressure, and MAP data for the attention deficit hyperactivity disorder (ADHD), alcoholism (ALC), cocaine addiction (COC), marijuana addiction (MAJ), and schizophrenia (SCH) studies, plus the matched controls (CTR).
(XLSX)
